# Aerosol delivery practice in Italian Cystic Fibrosis centres: a national survey

**DOI:** 10.1186/s40945-016-0015-3

**Published:** 2016-01-15

**Authors:** Simone Gambazza, Federica Carta, Anna Brivio, Carla Colombo

**Affiliations:** 1 Cystic Fibrosis Centre, Fondazione IRCCS Cà Granda Ospedale Maggiore Policlinico, Milano, Italy; 2 Associazione Riabilitatori dell’Insufficienza Respiratoria (ARIR), Milano, Italy

**Keywords:** Aerosoltherapy, Cystic fibrosis, Physiotherapy, Questionnaire, Education

## Abstract

**Background:**

Physiotherapists (PTs) are ideally positioned to assist patients and families with inhalation therapies through monitoring, communication and education about available therapies and their proper use; indeed aerosoltherapy management is listed as part of Italian PTs' core competence and in the core syllabus for post-graduate training in respiratory physiotherapy. The aim of this study was to outline the involvement of Italian PTs working in Cystic Fibrosis (CF) centres in the aerosol delivery practice.

**Methods:**

Physiotherapist coordinators (*n* = 29) of all Italian CF centres were invited to participate in a cross-sectional survey and a semi-structured questionnaire was developed and sent by e-mail.

**Results:**

A response rate of 69 % was achieved. The majority of participants were woman and the overall mean professional experience was twenty years. Italian PTs are involved in the aerosol delivery practice, managing education, drug-device combination, dilution and mixing of drugs.

**Conclusions:**

Physiotherapists play a key role in the care of Italian CF patients; nevertheless the Italian Group of Physiotherapists might plan interventions to harmonize the aerosol delivery practice in Italian CF centres and to sustain continuing education.

## Background

Effective administration of aerosolized medications depends on the patient’s age, physical and cognitive ability, the delivery system and the patient-device interface [1]. Many types of inhalation devices are available for use with various medications in Cystic Fibrosis (CF), however there are no clinical evidences about the supremacy of one device over another [1, 2] and few high-graded literature on the matter [3]. Physiotherapists (PTs) are ideally positioned to assist patients and families with inhalation therapies through monitoring, communication and education about the available therapies and their proper use. Several new drug formulations and new inhalation devices have been developed so far for CF thus circumventing the problem of poor penetration of intravenously administered antibiotics into lung parenchymal tissue and bronchial secretions, and their potential toxicity. Given that aerosoltherapy management is listed as part of the core competence of Italian physiotherapists working in CF [4] and aerosoltherapy is listed as well in the core syllabus for post-graduate training in respiratory physiotherapy [5], we aimed to outline the involvement of Italian PTs working in Cystic Fibrosis centres in the aerosol delivery practice, taking into account that Italy is the fourth European nation for number of people with CF (4119, European Registry 2010) and that these are followed-up in 22 referral regional centres and in 7 support centres by 93 physiotherapists as a whole, 29 of which are physiotherapist coordinators.

## Methods

All physiotherapist coordinators (*n* = 29) of Italian CF centres were invited to participate in a cross-sectional survey, and they were reached through the mailing list of the Italian CF Group of Physiotherapists (ICFGP) belonging to the Italian Society for the Study of Cystic Fibrosis. This study was conducted during January 2014 and data collection period lasted one week, with a gently reminder sent after four days.

The first step was a detailed review of international literature, which allowed the selection of four topics to be covered by the questionnaire. These four were then declined in 37 items: 32 closed-ended questions, of which 5 were ordinal-polytomous, 21 nominal-polytomous and 6 dichotomous; 2 open-ended questions and 3 double-entry tables. The first topic investigated about the professionals involved in the selection and/or administration of aerosoltherapy (questions 4 to 10); a second topic questioned about general awareness on different devices and their use (questions 11, 13 to 23); the third topic investigated dilutions and mixtures of drugs (questions 28, 30 and 31) and the last topic investigated timing of aerosoltherapy administration, evaluation of educational interventions and aerosol performance and tolerability as well (questions 12, 24 to 27, 29, 32 to 37).

To determine content-related validity, two external senior physiotherapists assessed face validity of the questionnaire and thus it was administered after two revisions. The final form of the questionnaire included an introductory paragraph on its first page outlining the main study aims, clarifying that participation was voluntary and assuring anonymity and confidentiality of information given. The questionnaire started with three questions about participants’ demographics and professional experience.

Data were analysed and presented as counts or percentages. One open-ended question regarded the participants’ job place if different from a referral or support CF centre; the second open-ended question was aimed to record the name of bronchodilator used as pre-medication during spirometry.

## Results

Twenty physiotherapist coordinators from 20 centres completed the survey. Overall response rate was 69 %, specifically 65 % for referral regional and support centres in the North of Italy. Seventeen centres (85 %) deal with both paediatric and adult patients, 2 centres (10 %) with adult and one centre (5 %) with paediatric patients only. Non-respondents work in 9 referral regional centres, 67 % in the South and 33 % in the North of the country. The majority of participants were woman (65 %); overall their mean professional experience was twenty years (ranging from 6 to 34 years).

All centres resulted to be familiar with all the available devices for aerosoltherapy in CF: ultrasonic, jet and vibrating mesh nebulizers, dry-powder inhalers (DPIs) and pressured metered dose inhalers (pMDIs) (soft mists were excluded); only two centres declared poor familiarity with ultrasonic nebulizers.

DPIs are mainly addressed to patients aged above 18 years (60 %) and 25 % of centres choose this device for patients aged 4 years or more. Eighteen centres advice pMDIs at any age (and 10 % use a spacer or a valved-holding chamber seldom) and two centres consider this device appropriate only for patients above 18 years old. The drug-device combination as matched by physiotherapists according to their practice is reported in Table 1.

**Table 1:**
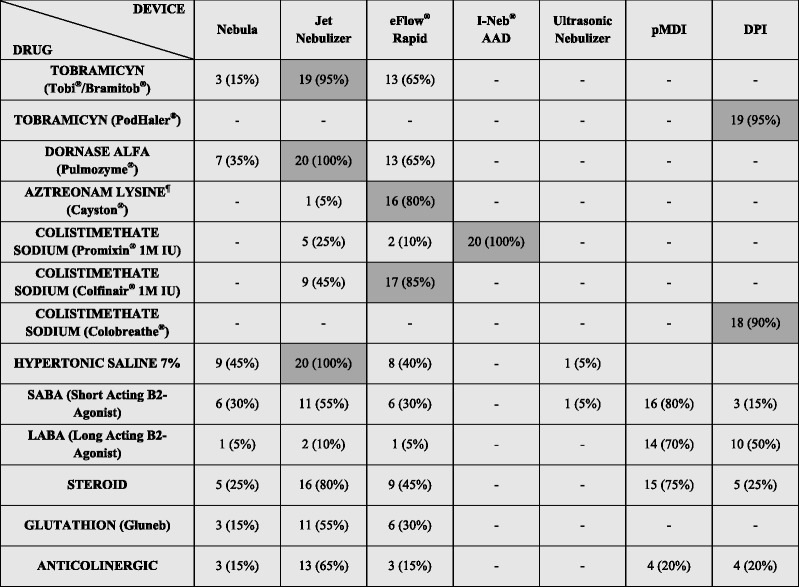
Drug–device combination as matched by physiotherapists during clinical practice

Dark-grey boxes show the suggested drug-device combination [10]. Percentages total can be more than 100 % because each respondent could choose more than one combination. ^¶^Four centres did not answer

PTs demonstrate how to use aerosoltherapy devices and educate to their proper use in 70 % of the centres. Physicians (10 %) and nurses (10 %) are involved as well, but always together with PTs; in one centre the physician alone is deputed to device training. Instructions for devices assembly are given by PTs in 16 out of 20 centres (80 %). PTs are also responsible of the device maintenance in 18 centres and in 95 % of centres PTs are the only ones in charge of patients and care givers’ education about each aspect of inhalation therapy. In one centre only, nurse works with physiotherapist to educate patients’ about aerosoltherapy.

Four and three centres out of twenty reported dilution of tobramycin and dornase alfa (Pulmozyme^®^) as a normal practice, respectively (Table 2). Aztreonam lysine (Cayston^®^) is administered in 35 % of centres with the vials of saline available as diluent within the package; Promixin^®^ 1 M IU is administered in 90 % of centres with I-Neb^®^ AAD system with 1 mL sterile water or sterile 0.9 % saline; Colfinair^®^ 1 M IU is diluted with 3 mL sterile 0.9 % saline in 50 % of centres; Gluneb has an heterogeneous administration.

**Table 2:**
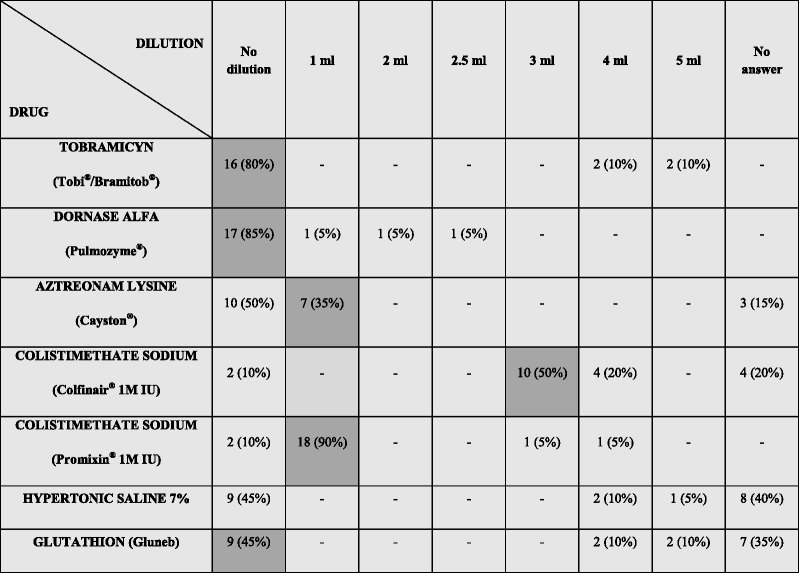
Drug–dilution practice as performed by physiotherapists in CF centres

Dark-grey boxes show suggested drug dilution, as reported in patient information leaflets and/or by pharmaceutical companies. Percentage total for Colistimethate sodium (Promixin^®^ 1 M IU) is more than 100 % because two centres gave more options

Mixing is not reported as a common practice in Italian CF centres: only 6 out of 20 centres allow mixing solutions and the combination of solutions mixed is reported in Table 3.

**Table 3:**
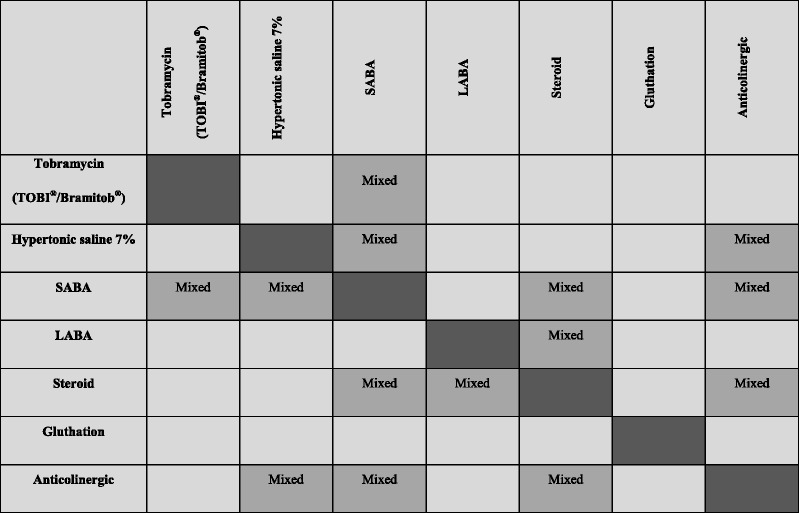
Inhalation solutions/suspensions allowed to be mixed in 6 out of 20 centres

SABA – short-acting beta_2_ agonist; LABA – long-acting beta_2_ agonist

As regards to the timing of inhalation therapy in relation to airway clearance techniques, almost all interviewees (80 %) put short-acting bronchodilators at the first place, followed by expectorants (65 %) before airway clearance therapies; inhaled antibiotics were placed afterwards (100 %), and 60 % of PTs suggested inhalation of dornase alfa after physiotherapy and 35 % before.

Testing inhalation tolerability with spirometry is a widespread practice in Italian CF centres: 10 % of them check response to new inhaled formulations with pulse-oximetry or pulmonary auscultation only. Spirometry is done before and after the first inhalation of a new formulation in 90 % of centres, and it is performed mostly by PTs (35 %), more rarely by pulmonary function technicians (10 %) and physicians (5 %). In the remaining half of the sample, spirometry is performed by any one of the professionals cited above. They all administer a short-acting beta_2_ agonist before inhalation, although no homogeneity was detected about the number of puffs delivered (from 2 to 4).

**Fig. 1:**
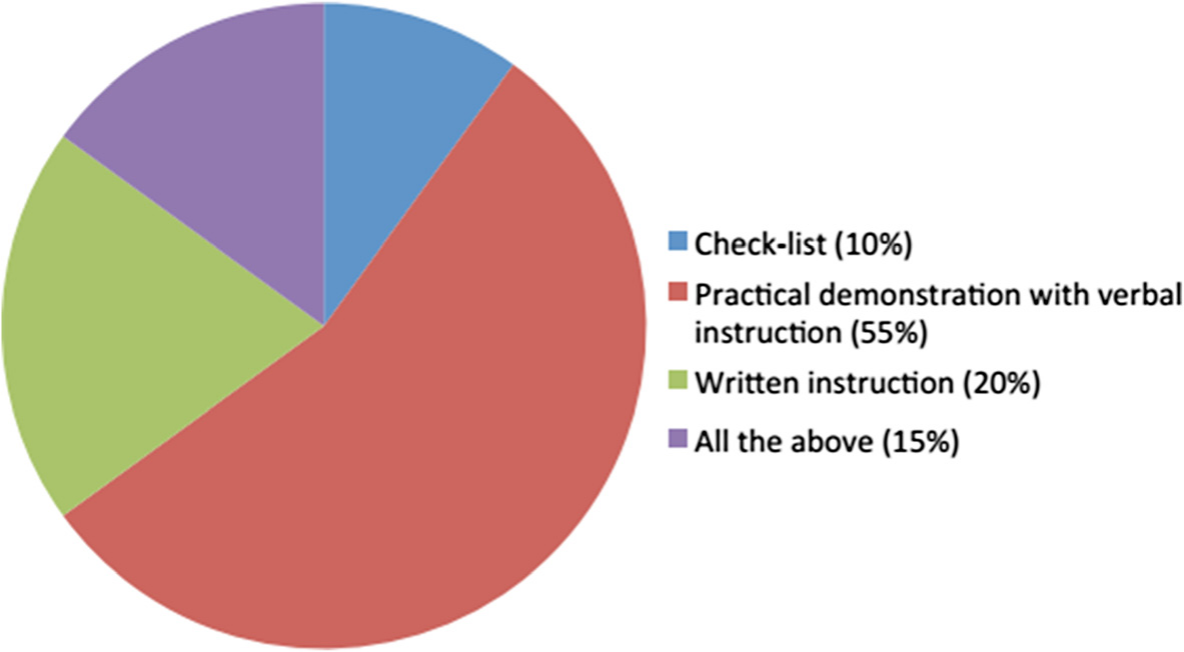
Educational strategies adopted by Physiotherapists

As far as training strategies, the majority of interviewees (55 %) take advantage of practical demonstrations (Fig. 1) and do not give patients nor care givers any written instruction. PTs are in charge of reviewing inhaled therapies and how they are administered in 90 % of CF centres, mostly during hospitalization.

## Discussion

People with Cystic Fibrosis have complex care needs that demand allied healthcare expertise. Indeed the life expectancy has increased significantly as a result of more effective treatments in specialized CF centres [6], where a specific knowledge and experience in the care of children and/or adults with CF is a key factor. A physiotherapist defined *CF specialist* is recommended for providing high-quality treatment of airway clearance, physical exercise and aerosoltherapy. Thus people with Cystic Fibrosis should be cared for by physiotherapists with an appropriate level of expertise in the physiotherapy management of CF and there should be adequate staffing levels to maintain these standards of care too (e.g. 2 PTs caring for 50 CF patients, 3 PTs caring for 150 patients or 4 PTs for ≥250 patients for a paediatric CF centre) [6]. These specific knowledge and skills required to PTs for dealing with respiratory diseases, such as CF, are just some of the contents of the training programme for respiratory physiotherapy as described by the Respiratory Physiotherapy HERMES (Harmonising Education in Respiratory Medicine for European Specialties) task force [5]. While the syllabus (available at http://ow.ly/zYkOD) lists the core competencies that practicing specialists in respiratory physiotherapy must possess, the respiratory physiotherapy HERMES task force is still currently working on a comprehensive curriculum, which provides a framework for the implementation of a post-graduate training programme in respiratory physiotherapy in individual countries. The advantage of fixed standards for training, assessment and institutional accreditation has the potential to safeguard practice and ensure quality care, pushing ahead the cultural and professional growth of the physiotherapy profession.

As outlined in the present survey, physiotherapists play a key role in the care of Italian CF patients, from performing inhaled therapies to educating patients and families to their use. These results are coherent with the role of physiotherapists involved in the respiratory care of Cystic Fibrosis as just outlined in the European Cystic Fibrosis Society standards of care [6], according to which < < As CF Physiotherapists are responsible for inhalation therapy they should be familiar with techniques, equipment provision and appropriate maintenance of devices> > .

Surprisingly, this survey revealed aspects such as right drug-device combination [7], correct dilution and mixing of drugs [8], which need to be urgently improved within the Italian CF Group of Physiotherapists.

There are nebulizers that may show a faster delivery time, but even the most effective medication cannot be delivered properly if wrongly administered or devices are not correctly cleaned. Variability reported in dilution practice is a matter of concern and each centre should discuss these differences and harmonize its own practice according to instruction given by pharmaceutical companies, keeping in mind that dilution of some drugs makes their inhalation useless or even harmful, hampering efficacy and adherence, as a result.

Half sample should review their educational strategies, considering that patients will experience better FEV_1_ with increased adherence to prescribed inhaled therapies [9]. It is recommended indeed that education to inhaled therapies is based on oral and written instructions [1, 2, 10], but only 55 % of Italian CF PTs use the latter. It is responsibility of the CF care team to routinely assess the educational needs of patients and family members and to correct incomplete or incorrect information using as many modalities as needed, such as educational brochures, links to online website [11] and new technology. A local “inhaled therapy coordinator” [9] should be identified to educate healthcare professionals and patients in addition to running periodic evaluations; it is suggested that patients should be evaluated one month after treatment has started and regularly assessed afterwards [7], at least annually by a CF specialist physiotherapist [10]. Even if a spirometry [4, 10], an assessment of breathlessness and sense of well-being [9] are the only measures suggested, especially if inhaled antibiotics, dornase alfa and hypertonic saline are administered [10], no guideline on the matter is available at the moment on lung function test practice in CF.

As the present study was designed as an electronic survey, there are important limitations concerning data interpretation: one week only was allowed for the completion of questionnaire and only physiotherapist coordinators were involved, maybe inaccurately representing the real practice of aerosol delivery as done in their centres.

## Conclusion

To our knowledge, this is the first time that involvement of physiotherapists in aerosoltherapy practice was investigated in Italian CF centres. The findings of the present study indicated the leading role of physiotherapists in aerosol delivery practice in the Cystic Fibrosis care but showed a worrisome and inhomogeneous behaviour among CF centres, requiring urgent revision.

The Italian CF Group of Physiotherapists might plan interventions to harmonize the aerosol delivery practice in CF centres and to sustain continuing education of physiotherapists and of all other professionals involved in fostering patients’ education. New drugs and devices are being placed on the market and it is one of the duties of CF physiotherapists to continue to deepen their knowledge and acquire new ones in this field.

## Abbreviations

CF: Cystic fibrosis
PTs: Physiotherapists
ICFGP: Italian cystic fibrosis group of physiotherapists
DPIs: Dry-powder inhalers
pMDIs: pressured metered dose inhalers
IU: International Units
AAD: Adaptive Aerosol Delivery
HERMES: Harmonising education in respiratory medicine for european specialties
FEV_1_: Forced expiratory volume in the first second
